# Medullary Thyroid Carcinoma With Elevated Serum CEA and Normal Serum Calcitonin After Surgery: A Case Report and Literature Review

**DOI:** 10.3389/fonc.2020.526716

**Published:** 2020-10-27

**Authors:** Li Chen, Ke Zhao, Fuxin Li, Xianghui He

**Affiliations:** Department of General Surgery, Tianjin Medical University General Hospital, Tianjin Medical University, Tianjin, China

**Keywords:** medullary thyroid carcinoma (MTC), carcinoembryonic antigen (CEA), calcitonin (Ctn), metastasis, management

## Abstract

**Background:**

Medullary thyroid carcinoma (MTC) is a relatively rare malignant tumor subtype originated the parafollicular C cells of the thyroid gland, producing tumor markers including calcitonin (Ctn), carcinoembryonic antigen (CEA), and chromogranin A. Preoperative serum Ctn and CEA value is important for assessing disease burden, postoperative serum Ctn and CEA can help to determine whether there are recurrence and distant metastasis.

**Case Presentation:**

We report a rare case in which the CEA level continued to increase and the Ctn value was normal after total thyroidectomy and central lymph node dissection in a MTC patient. The patient was asymptomatic during one and half year follow-up until lateral lymph node metastasis was revealed. However, the CEA level raised again after lateral neck lymph node dissection and bone metastases were found by 18F-FDG PET-CT.

**Conclusion:**

This case reminded us the recurrence of MTC should be suspected for patients with simply elevated CEA after surgery for MTC. Differential diagnosis of other malignant tumors and timely lymph node biopsy is of great significance for management.

## Background

Medullary thyroid carcinoma (MTC) originates from the thyroid follicle C cells that produce calcitonin, accounting for 5–10% of thyroid malignancy (1). MTC is divided into sporadically and hereditary form, as isolated familial MTC and multiple endocrine neoplasia type 2 (MEN 2), and mutation of RET proto-oncogene is the main molecular etiology. Over 50 years old, male gender, metastases, and MEN 2B type are considered poor prognostic factors. Sporadic MTC (SMTC) accounts for 80 to 90% in China, predominantly affecting adults aged 40–60, the clinical presentation of patients with SMTC is usually a palpable neck mass or cervical lymphadenopathy (2, 3). Local metastasis to cervical lymph nodes in the early stage of MTC, whereas hematogenous spreading to the liver, lung, or bone is not rare. MTC cells can produce multiple serum markers, including carcinoembryonic antigen (CEA), calcitonin (Ctn) and chromogranin A. The tumor has a unique characteristic expression of Ctn, which has been recommended by the guidelines as a serum marker for preoperative diagnosis of MTC, and postoperative monitoring to detect residual or recurrent disease. CEA and chromogranin A are also significant for the diagnosis of neuroendocrine tumors. More than 50% of patients with MTC have a mild elevation of CEA. Immunohistochemical studies have shown patients with more intensive staining for CEA are usually aggressive, disseminated MTC subtype (4). Ctn and CEA have high sensitivity and specificity in the preoperative diagnosis and postoperative follow-up of MTC patients, which have been recognized in clinical practice. Double time of serum calcitonin and CEA is also important prognostic indicators for patients with MTC (5), Ctn doubling time greater than 2 years is characteristic of indolent tumors, suggesting good long-term prognosis (6). However, in rare cases of MTC, Ctn levels were not correlated with the progress of disease and separation between serum Ctn and CEA levels were observed.

## Case Presentation

A 62 year-old woman was incidentally discovered with thyroid nodules by ultrasound. Neck examination revealed a hard painless nodule in the left lobe of thyroid with no palpable lymphadenopathy. No symptoms such as hoarseness, tachycardia, dyspnea, dysphagia, and weight loss. Family medical history was unsuspicious and no medical history of any malignancy. Ultrasonography revealed the thyroid nodule located in the deep outer layer of the left lobe, about 1.0*0.8*0.9cm in size, solid, hypoechoic nodule with irregular margins and calcified lesions inside, the aspect ratio of the nodule with visible blood flow signal was less than 1, and no lymphadenopathy, TI-RADS 4a; another cystic nodule of about 0.81*0.42 cm with regular margins and medium echo were seen in the deep lower layer of the right lobe, TI-RADS 3. Fine-needle aspiration (FNA) on thyroid prevailing nodule demonstrated indeterminate cytology. The pre-operative basal serum Ctn level was 41.10 pg/ml (normal values <5 pg/ml) and CEA level was 12.45 ng/ml (normal values <5 ng/ml) (06/10/2016). The patient did not perform a calcium stimulation test to determine calcitonin before surgery. Results of thyroid function tests, anti-thyroid antibodies, plasma catecholamine, and fractionated metanephrine were negative. Preoperative computed tomography of the chest and abdomen revealed no other extrathyroid lesions. The patient and her family refused the RET proto-oncogene genomic examination for economic reasons. Coincident phaeochromocytoma and primary hyperparathyroidism were excluded and the diagnosis of MTC was made. Total thyroidectomy and central compartment nodal dissection were performed.

**Figure 1:**
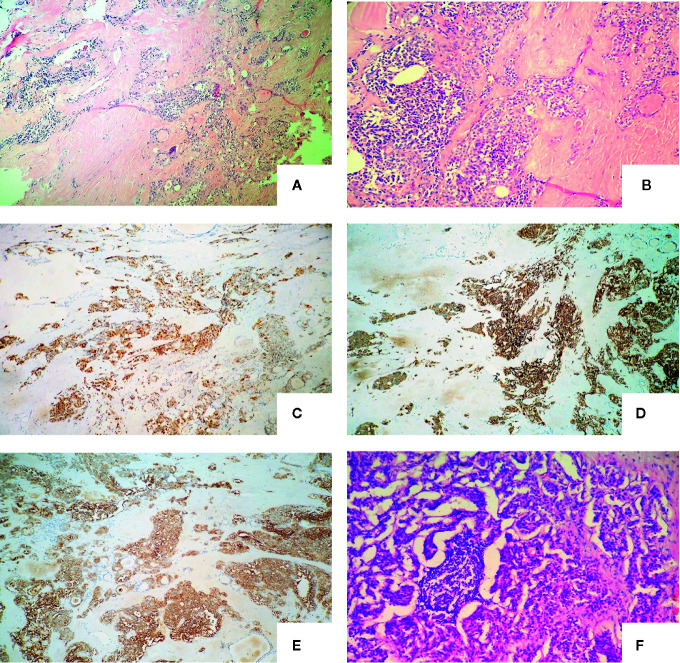
Definitive pathology of the thyroid tissue and lymph nodes of the first operation with the microscopy and immunochemistry evaluation: **(A)** The nests of neoplastic cells were separated by thick septa of fibrous tissue (haematoxylin and eosin, original magnification, x 100); **(B)** Amyloid deposits around the cell nest (haematoxylin and eosin, original magnification, x 200); **(C)** Neoplastic cells were strongly immunoreactive for chromogranin A (immunoperoxidase stain for anti-chromogranin A, original magnification, x 100); **(D)** Neoplastic cells were strongly immunoreactive for synaptophysin (immunoperoxidase stain for anti- synaptophysin, original magnification, x 100); **(E)** Neoplastic cells were strongly immunoreactive for carcinoembryonic antigen (immunoperoxidase stain for anti- carcinoembryonic antigen, original magnification, x 100); **(F)** Central lymph node metastasis. (haematoxylin and eosin, original magnification x 200).

**Figure 2:**
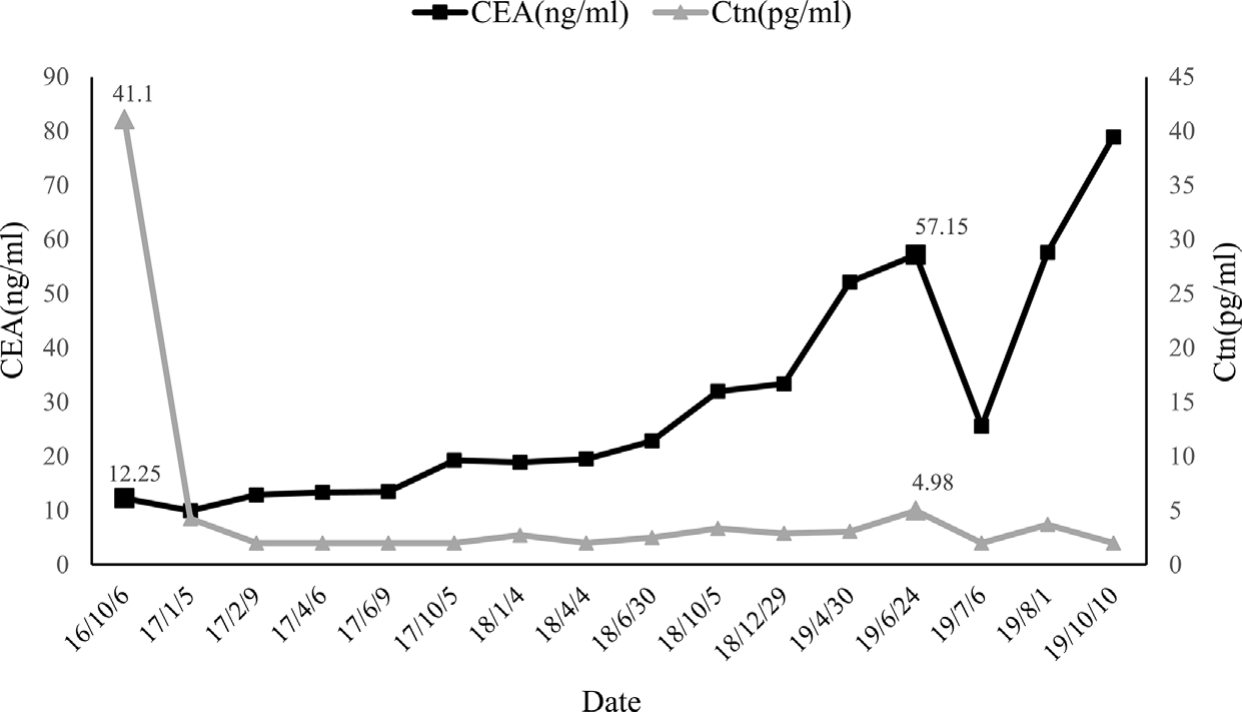
Changes in CEA and Ctn of serum levels from the fisrt operation to the last follow-up.

Intraoperatively, a 1.2*1.0cm hard mass was identified in the lower part of the left thyroid lobe, left hemithyroidectomy and isthmus resection was performed first and MTC was considered by fast-frozen section. Then completed thyroidectomy with lymphadenectomy of the central compartment as performing. Postoperative pathological findings revealed medullary carcinoma with no capsule invasion in the left lobe, and without any histological features of other malignant thyroid tumors including papillary thyroid carcinoma, follicular thyroid carcinoma, etc. Tumor cells were widely positive for CgA, Syn, CEA, Galectin-3, CK19, Ctn in immunohistochemistry. Partially positive for Tg, the Ki-67 index was about 1%. There was 1 out of 5 lymph node metastasis which originated from the primary lesions and had the same histomorphological pattern of the lesions in the central compartment with a cancer nodule detected (**Figure 1**). Serum calcium and parathyroid hormone values were normal after surgery and no surgery-related complications happened. Levothyroxine was administrated 100 ug daily after the operation to keep normal thyroid function. Three months after surgery (05/01/2017), the serum Ctn level was 4.25 pg/ml and the serum CEA level was 9.96 ng/ml, both of them were lower than that of preoperation. However, six months after the first operation, serum CEA levels increased consistently whereas Ctn levels remained normal as showed above (**Figure 2**).

**Figure 3:**
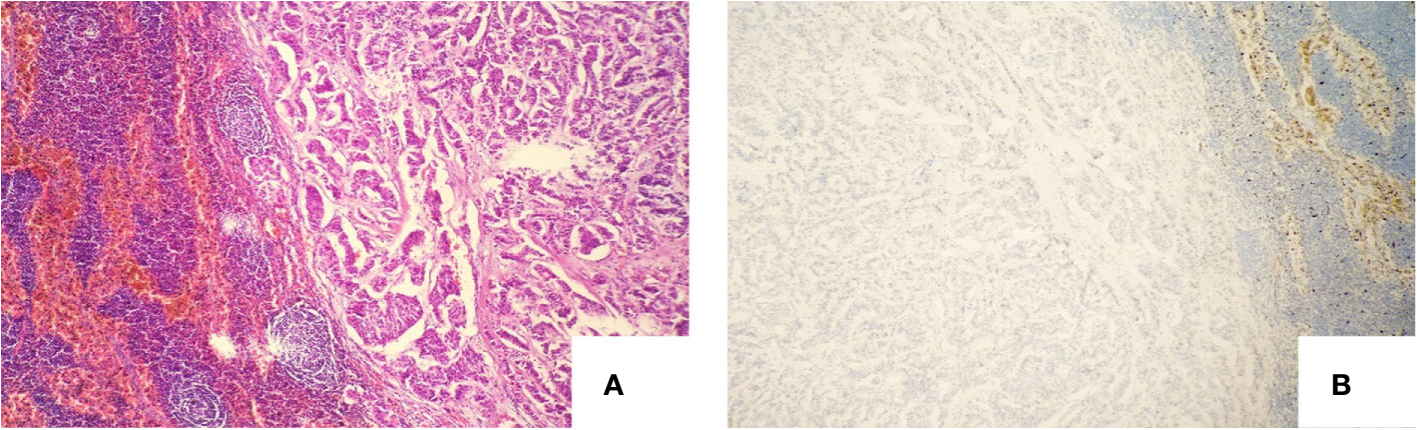
Definitive pathology of lymph nodes of the second operation with the microscopy and immunochemistry evaluation: **(A)** Recurrence of lateral cervical lymph nodes (haematoxylin and eosin, original magnification, x 100); **(B)** Neoplastic cells were negativity immunoreactive for calcitonin (immunoperoxidase stain for anti-calcitonin, original magnification, x 100).

The patient was asymptomatic during the follow-up. Due to the increased CEA levels, the patient underwent repeated neck ultrasonography, computed tomography of the neck, chest, and abdomen, pelvic MRI, gastrointestinal endoscopy, and total body 18F-FDG PET-CT. However, no positive results were found in all tests. Until one and a half years after the operation, ultrasonography revealed atypical lymph node with a size of 0.8*0.4cm in the left cervical region. FNA was performed and aspiration fluids were tested for CEA and Ctn. Laboratory results indicated CEA was 315.70 ng/ml and Ctn was over 2,000 pg/ml. Blood tests (24/06/2019) showed serum CEA was 57.15ng/ml and Ctn was 4.98 pg/ml. Cervical lymph node metastasis was considered and the patient was admitted for the re-operation. Cervical lymph node dissection was performed and a hard lymph node in the left neck-level IV was seen during operation. Postoperative pathology results showed lymph node metastatic neuroendocrine tumor (1/18) in level IV, which was consistent with the histopatological pattern of the primary lesions. Immunohistochemical staining showed positive for Syn, CgA, CK19, Galectin-3, and negative for Ctn and TPO. Lymph nodes in left neck level II, III, and IV showed no metastatic carcinoma (0/17, 0/5, and 0/7 respectively) (**Figure 3**). The patient recovered evenly without complications, the CEA values on days 1, 4, and 6 after the surgery was 36.24, 34.92, and 25.55 ng/ml, respectively. One month after the second surgery CEA levels raised to 57.64 ng/ml. Four months later, local bone destruction in the left scapula was found by total body 18F-FDG PET-CT, considering the possibility of distant metastasis, local bone destruction with a SUVmax of 3.6 in the left scapula was found by whole body 18F-FDG PET-CT, considering the possibility of distant metastasis. The latest CEA values of the patient without any symptoms were 78.95 ng/ml. It is recommended that patients continue to follow up, and no systematic treatment measures such as targeted therapy are currently available. 

## Discussion and Conclusion

MTC originates from parafollicular C cells of the thyroid gland which is thought to be derived from embryological neural crest (7). Clinical clues to MTC include a painful or painless solid nodule in the thyroid by palpation with elevated in serum Ctn and CEA. Regional metastases occur early in MTC, cervical lymph node metastases were founded in at least 50% of MTC cases, and distant metastases had occurred in 10 to 20% of MTC patients at the time of initial diagnosis (8). Lymph node metastases in MTC patients usually spread first to the central compartment, then to the ipsilateral lateral, and then to the contralateral lateral neck regions (9). Distant metastases to the lungs, liver, brain, skeletal system occurred in the late stages of the disease. Surgery is the only potentially curative method of treatment for MTC patients (10), total thyroidectomy and central node dissection is the primary surgical approach for management of the primary tumor of MTC (11). Ipsilateral II–V dissection confirms suspicious lymph nodes detected by ultrasound, CT, other imaging examinations, or intraoperative findings (12).

In general, elevated levels of CEA and Ctn in serum make them useful for initial diagnosis and long-term monitoring of disease status. Ctn (32-aminoacid polypeptidic hormone) is an ideal marker for MTC, serum Ctn sensitivity for diagnostic MTC is 98 to 99% (13), elevated basal or stimulated values of this peptide as a specific indicator is highly sensitive to the diagnosis of MTC. Machens and Dralle (14) also found a relation between preoperative basic Ctn values and lymph node involvement in various regions. MTC patients with lymph node metastases confirmed in the ipsilateral central region and lateral neck area, the contralateral central region, contralateral lateral neck, and superior mediastinum have increased their basic calcitonin thresholds by 20, 50, 200, and 500 pg/ml, respectively. These authors advocate that central and bilateral modified neck dissection can be performed with a preoperative basal Ctn value greater than 200 pg/ml.

MTC patients may find normal basal Ctn levels, preoperative basal Ctn negative MTC had been reported in the literature (15, 16). The deficient Ctn production might be related to the de-differentiation of tumor cells, which may be an indicator of poor prognosis (17, 18). However, this view was still controversial. A study which involved 839 patients suggested that the heterogeneous prognosis of Ctn negative MTCs ranges from long-term survival to rapid deterioration of lesions. Besides, the weak immunostaining of Ctn was not associated with aggressive behavior and poor prognosis (19). When combined with some of the following diseases including autoimmune thyroiditis, C-cells hyperplasia, hyperparathyroidism, some enteric and pulmonary neuroendocrine tumors, and chronic renal failure, it may cause serum Ctn levels to be incorrectly too low or too high. False-positive screening for Ctn results needs pentapeptide gastrin or calcium confirmatory stimulation tests, close follow-up, or diagnostic surgery to confirm (18). Ctn has also been suggested as a serum marker for postoperative monitoring to detect residual and recurrent lesions. Katerina Saltiki found that post-operative calcitonin is more important than size in predicting disease progression and prognosis through a retrospective study involving 128 patients with sMTC (≤ 1.5 cm) (20).

CEA is a non-organ specific tumor-associated antigen (adult normal value 0–5 ng/ml) and widely used in the diagnosis of malignant tumors such as a malignant gastric tumor, colorectal cancer, pancreatic malignancy, and malignant neoplasm of the respiratory system. However, more than 50% of patients with MTC have a mild elevation of CEA. It is reported in the literature that some patients with poorly differentiated MTC or metastasis may not have elevated calcitonin levels. Serum CEA is of great significance as a diagnostic biomarker for these patients (21). Immunohistochemical studies have shown patients with more intensive staining for CEA are usually aggressive, disseminated MTC subtype (4). Preoperative CEA values are associated with lymph node metastasis. Machens think that the continued increase in CEA levels indicates that MTC is in an advanced stage, the possibility of lymph node metastasis in the central and ipsilateral neck is high (22). When CEA >30.0 μg/l, the contralateral lymph node metastasis and distant metastasis are suggested when CEA >100 μg/l. However, the use of the CEA values to guide the region of secondary operation and decide the timing of the secondary surgery remains controversial for postoperative patients. Some scholars have suggested that neither preoperative nor postoperative CEA values were related to lymph node metastasis and CEA values should not be used as an indicator of the scope of surgery (16). Other experts confirm that the CEA level does not have the specificity of Ctn for MTC (10).

For postoperative MTC patients, the level of CEA and Ctn did not decrease compared with preoperative levels, which usually indicates that the primary lesion may be incompletely removed or there are potential ectopic lesions that secrete Ctn and CEA (17, 23). Mitra Niafar has reported increased calcitonin and CEA in a female MTC patient who underwent total thyroidectomy and central lymph node dissection. Finally, the ectopic lesions secreting CTn and CEA was found in the right side of anterior superior mediastinum by somatostatin receptor whole body scan examination (with 99mTc-HYNIC-TOC) (24). Similarly, the cases of persistence of detectable postoperative CT levels and normal CEA also can be seen in the clinical work. However, to our knowledge, this is the first report of medullary thyroid carcinoma with elevated serum CEA and normal serum calcitonin after surgery, and we emphasize that the case is extremely rare. The patient’s intralesional CEA and Ctn values was 315.70 ng/ml and over 2,000 pg/ml, respectively, but serum CEA was 57.15 ng/ml and Ctn was 4.98 pg/ml before the second operation in the case. The level of tumor markers in the lesion was significantly higher than that in the serology. The discrepancy between the serum and intralesional Ctn/CEA values suggested that the levels of tumor markers in the puncture fluid can be added for the postoperative MTC patient when needed during the follow-up period. Another interesting difference in the case was that the Ctn value tested on the relapse lesion exceeded 2,000 pg/ml, but the serum value was normal and Ctn immunohistochemical staining was negative. Calcitonin may be encapsulated in cells and not released into the blood or surrounding tissues, which leads to normal serum calcitonin levels. As for the calcitonin positive in the lesion but negative in immunohistochemistry, we consider that the calcitonin may be an incomplete fragment or propeptide of calcitonin, which cannot be recognized by immunohistochemical antibodies, but can be detected in the puncture fluid by ELISA. The increase of CEA value is related to MTC in the current case. No tumors of other potential organs and tissues were found during the follow-up period and before the second operation. Causes of elevated CEA were assessed thoroughly. Work-ups including neck ultrasound, computed tomography (CT) of the neck, chest, and abdomen, pelvic MRI, gastrointestinal endoscopy and whole body 18F-FDG PET-CT was performed to rule out the possibility of other malignant tumors. The diagnosis of MTC recurrence was not confirmed until an abnormal lymph node appeared and punctured. Four months after the second operation, whole body 18F-FDG PET-CT has performed again due to elevated CEA, metastatic lesions in the left scapula were revealed.

In summary, we reported a case of metastatic MTC with elevated CEA but normal Ctn. The diagnosis and management of metastatic MTC with simply elevated CEA are challenging before metastatic lesions are discovered.

## Data Availability Statement

All datasets for this study are included in the article.

## Ethics Statement

Written informed consent was obtained for the publication of any potentially identifiable images or data included in this article.

## Author Contributions

LC wrote the manuscript. KZ and FL contributed to the acquisition and analysis of the data and images of the patient. XH revised the manuscript critically for important intellectual content. All authors contributed to the article and approved the submitted version.

## Conflict of Interest

The authors declare that the research was conducted in the absence of any commercial or financial relationships that could be construed as a potential conflict of interest.

## Abbreviations

MTC, Medullary thyroid carcinoma; CEA, Carcinoembryonic antigen; Ctn, Calcitonin; FNA, Fine-needle aspiration; CT, Computed tomography; 18F-FDG PET-CT, 18F-flurodeoxyglucose positron emission tomography-Computed tomography.
